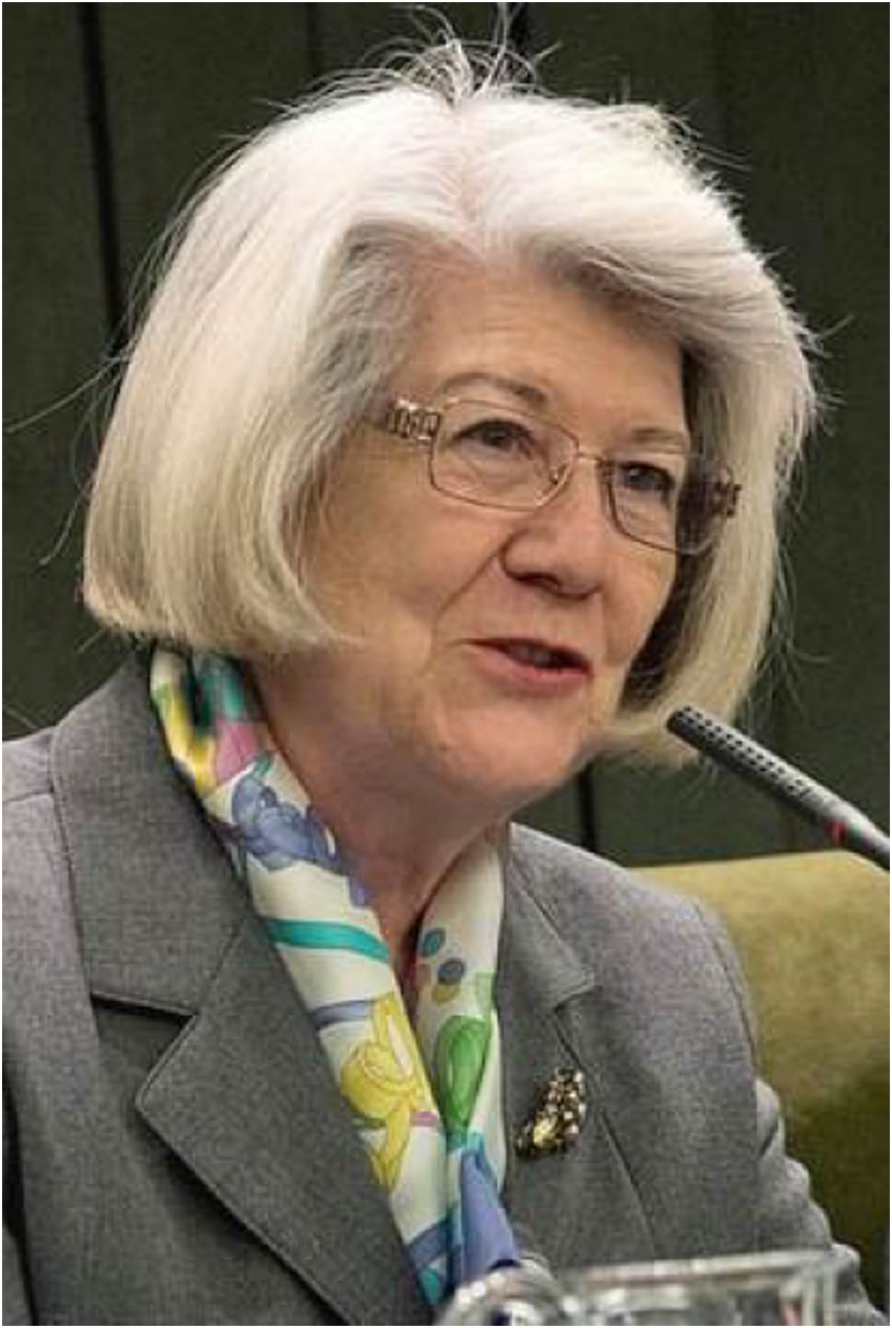# Maria Teresa Miras Portugal (1948–2021): in memoriam

**DOI:** 10.1007/s11302-021-09800-y

**Published:** 2021-07-01

**Authors:** Herbert Zimmermann, Simon C. Robson, Esmerilda Garcia Delicado, Maria Abbracchio, Francesco Di Virgilio, Alexej Verkhratzky, Carlos Matute

**Affiliations:** 1 Institute of Cell Biology and Neuroscience, Goethe University, Frankfurt am Main, Germany; 2 Beth Israel Deaconess Medical Center, Harvard Medical School, Boston, USA; 3 Departamento de Bioquímica Y Biología Molecular, Facultad de Veterinaria, Universidad Complutense de Madrid, Madrid, Spain; 4 Department of Pharmaceutical Sciences, University of Milan, Milan, Italy; 5 Department of Medical Sciences, University of Ferrara, Ferrara, Italy; 6 The University of Manchester, Manchester, UK; 7 Achucarro Center for Neuroscience, University of País Vasco, Leioa, Spain

The “Purinergic Signalling” community has lost one of its pioneers, fundamental pillars, and one of the most wonderful personalities. Maria Teresa was a scientist of exceptional creativity, drive, and radiance, an incredibly strong woman, and warm-hearted person. Unfortunately, Maria Teresa died far too early, following a protracted, serious illness leaving her family, friends, and colleagues around the world bereft. She was vibrant and so full of life in 2019, when we had gathered at the 1st European Purine Meeting in Santiago de Compostela, which she had organized. That she is no longer with us is hard to believe but we will be able to pay tribute to her in a future issue of “Purinergic Signalling.”

Born in 1948 in the small Galician town of O Carballiño, she studied Pharmacy at the University of Santiago de Compostela and at the Complutense University Madrid. For PhD studies, she went to Strasbourg (France) to work with Dominique Aunis and Paul Mandel in the Centre de Neurochimie du CNRS (1975). She had moved there with her husband and her first-born son, Fernando, and her second son, Alberto, was born there during her doctoral studies.

Her extensive, innovative studies first concerned the characterization of dopamine-ß-hydroxylase in the bovine adrenal medulla. Chromaffin cells then served as her model cellular system for many years, albeit using different experimental approaches. Her studies led to Professorships in Oviedo and Murcia. In 1986, she took the chair and directorship of the Departamento de Bioquímica y Biología Molecular IV, Facultad de Veterinaria of the Complutense University Madrid.

Maria Teresa made important contributions to many fields of purinergic signaling. She turned to these experimental systems early on, studying glucose transport, adenosine uptake, and purine nucleotide synthesis in chromaffin cells. Based on these studies, in 1988, she demonstrated storage of diadenosine polyphosphates in chromaffin granules. She became *the* pioneer in the field studying the biology of dinucleoside polyphosphates. Detailed quantitative studies followed to characterize uptake and release of dinucleoside polyphosphates as well as the extracellular hydrolysis by chromaffin cells of nucleotides, particularly of diadenosine polyphosphates. She demonstrated that diadenosine polyphosphates activate P2 receptors and described the physiological activity of these mediators in the central nervous system and other tissues. Moreover, the roles of tissue-nonspecific alkaline phosphatase in neuronal development, the associated signaling pathways via dual specificity protein phosphatases in neurons, and glial cells as well as the vesicular nucleotide transporter became major interests of her group.

Towards the end of the 1990s, she increasingly focused on experimental models involving purinergic receptors in the central nervous system. Early on, she recognized the relevance of the purinergic signaling pathway in disease and realized the potential for therapeutic approaches. Animal models of disease came more and more to the fore in her laboratory: providing insights into Huntington’s chorea; Alzheimer’s disease; neuroprotection; neuroregeneration, and status epilepticus. Her detailed investigations shed important new light in particular on P2X7 and P2Y_13_ receptors and the respective intracellular signaling cascades. Her last comprehensive review article, published in 2021, focused on the P2X7 receptor.

Maria Teresa was heavily involved in the development of international science. Legendary are the exceptionally well ran conferences she organized. Amongst these was the landmark “Purines 2000,” an international conference on nucleotides and adenosine held in Madrid, at the time of this field enjoying major growth and development. Thereafter were several wonderful, memorable conferences on select topics of purinergic signaling, generously supported by the Ramón Areces Foundation. The last time we could enjoy her exceptional hospitality was in September 2019 in Santiago de Compostela, at the “1st European Purine Meeting.” This conference was designed to replace the traditional biannual Italian/German–German/Italian Purine Club meetings and took these to a higher international level. In Santiago de Compostela, she was instrumental in launching the “European Purine Club” which was to be developed further at the next meetings. She would and should have been the first president of this grouping. Her engagement in the European and International research community was exemplified also by the regular mutual exchange of PhD students and postdoctoral fellows with several other countries around the globe. In this respect, she has been an example for young women interested in pursuing a scientific carrier, demonstrating that you can be a solid and motivated woman scientist without giving up having a family and personal life. She has always strongly supported young women scientists’ careers by encouraging them to foster their research interests and by offering opportunities to start collaborations with other laboratories.

Yet, these were only a few aspects of Maria Teresa’s multiple involvements and talents. From 2007 to 2013, she served as president of the prestigious “Real Academia Nacional de Farmacia,” where she provided important stimuli for innovations. She was the first woman to be elected president of any of the Spanish Royal Academies. Following her tenure with its impressive impact, she was elected honorary president. She held membership of several Spanish scientific institutions, was on the editorial boards of many international journals, on the IUPHAR sub-committee for the nomenclature of P2Y receptors, and held position on the Scientific Panel of NATO.

Her multiple activities in science and culture were honored by several prestigious awards. These include the “Alberto Sols Medal to Research in Biochemistry,” the “María Josefa Wonenburger Planells Prize,” a prize provided by the Galician government and University of A Coruña, which recognizes women with exceptional achievements in science and technology, and the “Castelao Medal in Galicia,” an award presented by the Galician government to honor people and institutions with exceptional works in the arts, culture, literature, and science. The Community of Madrid honored her with the “Miguel Catalonian National Research Award” in recognition of her entire professional career.

She also received honorary doctorates from the Catholic San Antonio University of Murcia (UCAM) and the University Rey Juan Carlos Madrid. She was member of the “Academia Nacional de Farmacia y Bioquímica de Argentina,” “Academie Nationale de Pharmacy de France,” “European Academy of Science, Arts and Humanities,” and the “Academia Europaea (Physiology and Neuroscience).” She was an extraordinary person with great passion for science and life. She was held in extraordinary regard by young scientists and trainees. We shall remember Maria Teresa as an excellent scientist, a brilliant, creative intellectual, and as a woman of great strength, passion, and empathy. Her career and life can be considered exemplary in all respects. She will continue to serve as an inspiration to our Spanish-speaking scientific community, indeed for all of us, and especially to young women scientists all around the world.